# Translation fidelity coevolves with longevity

**DOI:** 10.1111/acel.12628

**Published:** 2017-07-13

**Authors:** Zhonghe Ke, Pramit Mallik, Adam B. Johnson, Facundo Luna, Eviatar Nevo, Zhengdong D. Zhang, Vadim N. Gladyshev, Andrei Seluanov, Vera Gorbunova

**Affiliations:** ^1^ Department of Biology University of Rochester Rochester NY USA; ^2^ Instituto de Investigaciones Marinas y Costeras CONICET‐UNMdP Mar del Plata Argentina; ^3^ Institute of Evolution University of Haifa Haifa 3498838 Israel; ^4^ Department of Genetics Albert Einstein College of Medicine Bronx NY USA; ^5^ Division of Genetics Department of Medicine, Brigham and Women's Hospital Harvard Medical School Boston MA USA

**Keywords:** aging, comparative biology, longevity, translation fidelity

## Abstract

Whether errors in protein synthesis play a role in aging has been a subject of intense debate. It has been suggested that rare mistakes in protein synthesis in young organisms may result in errors in the protein synthesis machinery, eventually leading to an increasing cascade of errors as organisms age. Studies that followed generally failed to identify a dramatic increase in translation errors with aging. However, whether translation fidelity plays a role in aging remained an open question. To address this issue, we examined the relationship between translation fidelity and maximum lifespan across 17 rodent species with diverse lifespans. To measure translation fidelity, we utilized sensitive luciferase‐based reporter constructs with mutations in an amino acid residue critical to luciferase activity, wherein misincorporation of amino acids at this mutated codon re‐activated the luciferase. The frequency of amino acid misincorporation at the first and second codon positions showed strong negative correlation with maximum lifespan. This correlation remained significant after phylogenetic correction, indicating that translation fidelity coevolves with longevity. These results give new life to the role of protein synthesis errors in aging: Although the error rate may not significantly change with age, the basal rate of translation errors is important in defining lifespan across mammals.

## Introduction

The error catastrophe theory of aging was proposed by Orgel in the 1960s (Orgel, 1963, 1970, 1973). According to this model, the aging process results from errors in mRNA translation that reduce the fidelity of the protein‐translating enzymes leading to increasingly inaccurate protein synthesis, terminating in functional decline, and, ultimately, the death of the organism (Srivastava & Busbee, 2003). This theory, for the first time, proposed that translation fidelity plays a major role in aging.

The error catastrophe theory has been challenged by a number of studies in the 1980s (Edelmann & Gallant, 1977; Wojtyk & Goldstein, 1980; Mori *et al*., 1983; Szajnert & Schapira, 1983). For instance, human cultured fibroblasts did not show an increase of translation errors at late passages (Wojtyk & Goldstein, 1980), tyrosine aminotransferase purified from livers of old rats did not show altered physicochemical properties (Szajnert & Schapira, 1983), and ribosomes from mouse livers did not show changes in codon misrecognition at the first and second positions during aging (Mori *et al*., 1983), suggesting that translational errors do not increase significantly during the aging process. A major caveat of these studies, however, is that many of them were conducted *in vitro* following ribosome isolation. As the aging process affects entire cellular networks, isolated proteins or other cellular components may not fully recapitulate this *in vivo* process. Interestingly, a theoretical study using molecular level evolutionary simulations suggested that mistranslation‐induced protein misfolding imposes strong evolutionary pressure especially in the neural tissues (Drummond & Wilke, 2008).

During translation initiation, the small and subsequently large ribosomal subunits bind to the mRNA. The ribosome carries three tRNA binding sites: the aminoacyl (A) site, the peptidyl (P) site, and the exit (E) site. In the translation elongation stage, a ternary complex composed of the elongation factor eEF1A (EF‐Tu), aminoacyl‐tRNA, and GTP is deposited into the A site. Next, peptidyl‐transfer takes place, where the carboxyl end of the polypeptide chain is released from the tRNA at the P site and joined to the free amino group of amino acid linked to the tRNA at A site. To complete the cycle, the peptidyl‐transfer reaction is followed by translocation into the E site and P site, leaving the A site ready for the next round of elongation (Zaher & Green, 2009a). Quality control mechanisms operate at many steps of translation (Reynolds *et al*., 2010). Aminoacyl‐tRNA synthetases maintain fidelity during protein synthesis by attaching amino acids to their cognate tRNAs (Ling *et al*., 2007). Some of the aminoacyl‐tRNA synthetases possess an editing activity that hydrolyses the bond between incorrectly ligated amino acid and tRNA. The tRNA synthetases’ editing activity is important for the fidelity control (Ling *et al*., 2007). An additional level of protection may be conferred by discrimination by elongation factor eEF1A (EF‐Tu) against misacetylated aminoacyl‐tRNAs (LaRiviere *et al*., 2001). The aminoacylation step has been shown to be very accurate; therefore, *in vivo* the fidelity is believed to be largely determined by the mistakes in the decoding of the mRNA by the ribosome. During initial codon pairing at the A site, the rate of GTP hydrolysis by the aminoacyl‐tRNA–eEF1A–GTP complex is faster for a correct codon: anticodon pairing than an incorrect pair, increasing the probability that the incorrect complex will dissociate (Gromadski & Rodnina, 2004; Schmeing *et al*., 2009). Many tRNAs contain post‐transcriptional modifications in their anticodons, which can influence wobble during codon recognition and contribute to the accuracy of translation (Muramatsu *et al*., 1988). After GTP hydrolysis and eEF1A (EF‐Tu) release, there is the second proofreading step by the ribosome where the aminoacyl‐tRNA is more likely to be rejected if there is a mismatch between the codon and anticodon. After the peptide bond is formed and the newly formed peptidyl‐tRNA in the A site is translocated to the P site, a mismatched codon: anticodon pair in the P site leads to general loss of specificity in the A site of the ribosome, resulting in premature termination of elongation (Zaher & Green, 2009b, 2010).

Experimental models where translation fidelity was experimentally perturbed displayed shortened lifespan and susceptibility to disease. For example, mutations in tRNA genes and tRNA processing enzymes have been linked to various human diseases (Abbott *et al*., 2014), and phenylalanyl‐tRNA synthetase mutation in a Drosophila model has low translation fidelity and shortened lifespan (Lu *et al*., 2014). In addition, ribosomal RNA methyltransferase linked to translational fidelity modulates yeast replicative lifespan (Schosserer *et al*., 2015). It was also found that less efficient translational fidelity control was associated with tumor progression (Belin *et al*., 2009), and accumulation of mistranslated and misfolded proteins was implicated in several age‐related neurodegenerative diseases (Olzmann *et al*., 2008; Taylor & Dillin, 2011; Ciechanover & Kwon, 2015). These studies underscore the importance of translation fidelity for maintaining organismal health. However, to prove that a process controls aging and longevity, ideally one would have to improve this process and show that it leads to lifespan extension.

In the last decade, it was established that modulating the translational machinery can extend lifespan in a variety of organisms (Steffen & Dillin, 2016). Inhibition of the highly conserved target of rapamycin (TOR) pathway by mutations or chemical inhibitors such as rapamycin results in downregulation of protein synthesis and lifespan extension. Mutation or depletion of ribosomal proteins and translation factors leads to lifespan extension in yeast (Kaeberlein *et al*., 2005; Steffen *et al*., 2008), worms (Curran & Ruvkun, 2007; Hansen *et al*., 2007; Syntichaki *et al*., 2007), and flies (Kapahi *et al*., 2004; Pan *et al*., 2007), while rapamycin treatment extends lifespan of mice (Harrison *et al*., 2009; Selman & Partridge, 2012). The mechanisms explaining the life‐extending effects of TOR inhibition are not fully understood, but most evidence points toward preferential translation of specific transcripts involved in stress response, rather than improved fidelity of translation. This leaves an open question whether translation fidelity plays a role in aging, and whether it is possible to improve translation fidelity.

A study by our group showed that the longest lived rodent, the naked mole rat (NMR) has significantly increased translational fidelity in comparison to a short‐lived mouse (Azpurua *et al*., 2013). Interestingly, we found that the NMR 28S ribosomal RNA is cleaved into two fragments in mature ribosomes, which is unique among mammals and may be responsible for improved fidelity of protein synthesis in this species (Azpurua *et al*., 2013; Fang *et al*., 2014).

**Figure 1:**
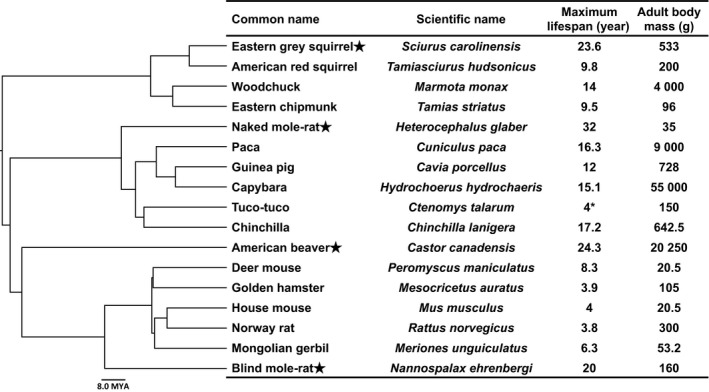
Phylogenetic tree and maximum lifespan data for the rodents used in this study. The lifespan and body mass data were derived from AnAge database (Tacutu *et al*., 2013). Stars indicate species with the maximum lifespan greater than 20 years. * For tuco‐tuco lifespan in the wild is shown, and maximum lifespan in captivity is not available.

To examine the role of translational fidelity in aging, we tested whether translational fidelity co‐evolved with species maximum lifespan. We examined translation fidelity in rodent species with diverse maximum lifespan ranged from 4 to 32 years (Fig. 1). We found a strong correlation between the frequency of mistranslating the first and second codon positions and the maximum lifespan in 16 rodent species. This correlation remained significant after phylogenetic correction by the method of independent contrast, indicating that translation fidelity co‐evolved with longevity. The fidelity of mistranslation at the third position and the misreading of a stop codon did not correlate with maximum lifespan, possibly due to the wobble effect at the third codon position, and to extremely low frequency of misreading the stop codon in all species. These results provide evidence that translation fidelity is an important factor in determining species lifespan.

## Results and discussion

**Figure 2:**
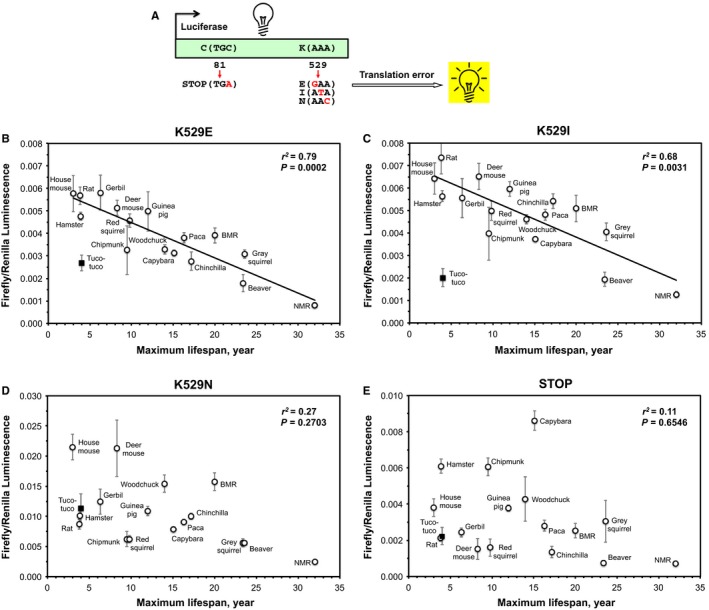
Translation fidelity correlates with species maximum lifespan. (A) The assay used to measure translation fidelity. The reporters contain firefly luciferase with mutations in K529, the amino acid essential for luciferase activity (Kramer & Farabaugh, 2007). Translation errors at this position lead to restoration of luciferase gene. The three reporters contain mutations in the first, second, and third codon positions and a stop codon at the amino acid position 81. The error frequency was calculated as the ratio of firefly luciferase to the Renilla luciferase, transfection control, the lower the ratio, the higher the fidelity. Primary, low passage fibroblasts were cotransfected with the firefly reporter constructs and Renilla luciferase. Three independent cell lines were assayed each species, and each cell line was assayed in triplicate. Error bars show SEM. BMR, blind mole rat; NMR, naked mole rat. (B) Misincorporation at the first codon position (K529E) shows strong negative correlation with maximum lifespan. (C) Misincorporation at the second codon position (K529I) shows strong negative correlation with maximum lifespan. (D) Misincorporation at the third codon position (K529N) does not significantly correlate with maximum lifespan. (E) Skipping of the STOP codon shows no significant correlation with maximum lifespan.

To measure the fidelity of protein synthesis, we used sensitive luciferase‐based reporters that we previously developed (Azpurua *et al*., 2013) (Fig. 2A). In these reporters, the firefly luciferase gene is mutated at position K529, the residue essential for luciferase activity (Kramer & Farabaugh, 2007). The mutations include nucleotide substitutions at the first (K529E), second (K529I), and third (K529N) codon positions and also a substitution to a stop codon (TGA) at the amino acid position 81. Amino acid misincorporation at these positions results in reactivation of the luciferase gene and is scored using a luminometer. Primary, low passage, rodent fibroblasts from 17 species with diverse lifespans from our collection (Seluanov *et al*., 2008, 2010; Patrick *et al*., 2016) were cotransfected with the luciferase reporters and Renilla luciferase as a transfection control. Three independent (derived from different animals) cell lines were assayed for each species, and each cell line was tested in triplicate.

We found a strong negative correlation between the frequency of errors at the first (*r* ^2^ = 0.79, *P *=* *0.0002) and second (*r* ^2^ = 0.69, *P *=* *0.0031) codon positions and species maximum lifespan (Fig. 2B, C). Long‐lived species such as beaver, gray squirrel, and NMR showed low frequency of errors, whereas short‐lived species, such as mouse, rat, hamster, gerbil, deer mouse and guinea pig, had lower translation fidelity. The frequency of translation errors did not correlate with body mass, neither for the first (*r* ^2^ = 0.15, *P *=* *0.6) nor for the second (*r* ^2^ = 0.12, *P *=* *0.69) codon positions (Figure S1A, B). However, as body mass was previously shown to correlate positively with longevity, to control for the effects of possible correlations between body mass and lifespan, we studied the independent contribution of each to the translation fidelity by analyzing independent contrasts with multiple regression. Only maximum lifespan was significantly related to translation fidelity (*F* = 11.1, *r* ^2^ = 0.78, *P* < 0.001; body mass, *t* = −0.66, *P* = 0.52; maximum lifespan, *t* = −4.19, *P* < 0.001). This correlation between the translation fidelity and lifespan remained significant after phylogenetic correction by the method of independent contrasts (Felsenstein, 1985) (*r* ^*2*^ = 0.64, *P *=* *0.0002) for the first codon position and (*r* ^2^ = 0.25, *P *=* *0.04) for the second codon position, indicating that high fidelity of translation coevolved with lifespan in rodents.

The frequency of errors in reading the third codon position was much higher and did not significantly correlate with maximum lifespan (Fig. 2D) (*r* ^2^ = 0.27, *P *=* *0.2703) or body mass (*r* ^2^ = 0.12, *P *=* *0.68) (Figure S1C). The third codon position is known to display the “wobble effect,” which makes the pairing of codon and anticodon less stringent in the ribosome (Ogle & Ramakrishnan, 2005). This may result in low selection pressure for the accurate reading of the third codon position.

We also assayed frequency of mistranslating the stop codon. The frequency of these events was lower in the majority of the species and did not show significant correlation with maximum lifespan (Fig. 1E) (*r* ^2^ = 0.11, *P *=* *0.65) or body mass (*r* ^2^ = 0.08, *P *=* *0.76) (Figure S1D). Recognition of the stop codons is provided by the release factors, instead of tRNAs in case of amino acid insertion. Recognition of stop codons appears to have high fidelity regardless of species lifespan. This suggests that stop codon recognition is under high selective pressure, possibly reflecting the high energetic cost of failing to recognize a stop codon.

As the NMR has the highest translation fidelity and the longest lifespan, we further analyzed the correlations with the NMR omitted to test whether this one species drives the correlation. Without the NMR, we still found a significant negative correlation between the frequency of errors at the first (*r* ^2^ = 0.69, *P *=* *0.004) and second (*r* ^2^ = 0.53, *P *=* *0.044) codon positions and species maximum lifespan. The frequency of translation errors did not correlate with body mass, neither for the first (*r* ^2^ = 0.48, *P *=* *0.07) nor for the second (*r* ^2^ = 0.43, *P *=* *0.11) codon positions. The frequency of errors at the third (*r* ^2^ = 0.15, *P *=* *0.60) position and frequency of mistranslating the stop codon (*r* ^2^ = 0.04, *P *=* *0.88) did not correlate with species maximum lifespan after removal of the NMR. The frequency of errors at the third (*r* ^2^ = 0.007, *P *=* *0.77) position and frequency of mistranslating the stop codon (*r* ^2^ = 0.69, *P *=* *0.69) did not correlate with body mass either after removal of the NMR. We conclude the positive correlation between translation fidelity and lifespan in the first two codon positions remains significant even when the NMR is excluded.

The high translation fidelity in the NMR was in agreement with our earlier report that this species has about 10‐fold higher translation fidelity than the mouse (Azpurua *et al*., 2013). The NMR is one of only two mammals known to have split 28S ribosomal RNA (Azpurua *et al*., 2013; Fang *et al*., 2014). Remarkably, the only other known mammal with the split 28S rRNA, the tuco‐tuco (Melen *et al*., 1999), also showed high translation fidelity (Fig. 2). As the maximum lifespan is not established for the tuco‐tuco, we did not include this species in the correlation analysis. This result suggests that the uniquely split 28S rRNA structure may have evolved to increase the fidelity of translation in these two rodent species. However, the mechanism by which this unique 28S rRNA structure may confer higher translation fidelity is not clear. The cleavage of 28S rRNA leads to a deletion of the major part of the D6 region corresponding to a helix 45 of 23S rRNA in *E. coli* leaving the two fragments of 28S rRNA disconnected. This may change the folding or dynamics of the large ribosomal subunit, altering the rate of GTP hydrolysis by elongation factor 1A and/or interaction of the large subunit with tRNA during accommodation, thus affecting the fidelity of translation. In bacterial ribosome, helix 45 of 23S rRNA is connected to the L11‐stalk (L12 stalk in mammals), which is formed by helices 42, 43, and 44 through coaxial stacking with helices 40 and 41. Thus, the deletion of helix 45 may affect dynamics of the L11 stalk, which directly interacts with the elbow of the aminoacyl‐tRNA in the intermediate (A/T) state of tRNA accommodation (Schmeing *et al*., 2009). Future structural studies such as crystallography or cryo‐EM analysis of naked mole rat and tuco‐tuco ribosomes may shed light on the unique ribosomal organization in these species.

As the phylogenetic analysis has shown, the high translation fidelity has evolved independently in the long‐lived species. Thus, the mechanisms responsible for the high fidelity are expected to be different in different species. For example, the tuco‐tuco has evolved a single cleavage site within in 28S rRNA D6 region (Melen *et al*., 1999), while the NMR has independently evolved two cleavage sites within the 28S rRNA D6 region, resulting in a fragment being cut out (Azpurua *et al*., 2013). Both of these species may possess additional mechanisms of translational fidelity described below.

The other long‐lived species with high translation fidelity such as beaver and gray squirrel, have the conventional 28S rRNA structure, and may have evolved different mechanisms to increase translation fidelity. These mechanisms may include higher fidelity of aminoacyl‐tRNA synthetases, increased fidelity of base paring between codon: anticodon at the ribosome, higher fidelity of eEF1A, or higher efficiency of ribosomal proofreading. Different combinations of these mechanisms might have evolved in each of the long‐lived species. Currently, the mechanisms responsible for maintaining translation fidelity are best understood in prokaryotes or lower eukaryotes. Deeper understanding of protein translation in mammals is needed to begin addressing the mechanisms responsible for interspecies differences in translation fidelity among mammals.

The luciferase‐based assay may, in principle, detect errors in transcription, in addition to errors in translation. However, several lines of evidence argue against this possibility. First, error rates in transcription are about an order of magnitude lower than those in translation (Milo & Phillips, 2015) and therefore would not significantly contribute to the results. Second, there is a clear difference between the error rates for the misreading of the first two and the third codon positions. RNA synthesis errors would not be expected to show such a bias. Third, much higher fidelity of stop codon recognition is again a characteristic of protein synthesis and would not be expected for the RNA synthesis error.

We identified that the frequency of mistranslation correlates negatively with lifespan by monitoring misincorporation of two near‐cognate codons in the first and second positions. Studies in *E. coli* (Kramer & Farabaugh, 2007) and yeast (Plant *et al*., 2007) showed that rare codons have higher propensity of mistranslation due to low frequency of the corresponding tRNAs. As our study was limited to two relatively frequent codons, we do not know whether misincorporation of other codons would correlate with lifespan. However, because codon usage is very similar among mammalian species, we predict other codons would show the same trend.

The cells in our study were derived from young adult animals. Wild rodents, being prey animals, do not survive to advanced age in the wild, and it is not possible to know the precise ages of wild caught animals. Therefore, our study did not examine the change in translation fidelity over an individual lifespan but rather compared the basal rates of translation errors among the young adult individuals of different species.

Earlier studies did not find evidence for increased rate of errors in protein synthesis during organismal aging (Edelmann & Gallant, 1977; Wojtyk & Goldstein, 1980; Mori *et al*., 1983; Szajnert & Schapira, 1983). It is possible that errors in protein synthesis impose selective pressure on the evolutionary scale (Drummond & Wilke, 2008), but not during individual lifespan. Alternatively, these earlier studies may have had low sensitivity to detect such a change. Most of these studies were conducted *in vitro* on isolated proteins and used less sensitive assays. *In vivo* studies using sensitive reporters are needed to test whether translation fidelity changes during aging of an organism.

In summary, we have demonstrated that translation fidelity co‐evolves with longevity. In longer‐lived species mechanisms have evolved to make translation more accurate. A lower rate of errors in protein synthesis may contribute to fewer aberrant and aggregated proteins and reduce lifetime proteotoxic stress. Our study does not directly support the protein catastrophe theory of aging, but demonstrates that the fidelity of protein synthesis is a determining factor in the evolution of lifespan.

## Methods

### Cell culture

Primary rodent fibroblasts were from our established collection (Seluanov *et al*., 2008). Cells were cultured as described (Seluanov *et al*., 2010). Briefly, fibroblasts were cultured at 37 °C, 5% CO_2_, and 3% O_2_ incubator, except the NMR fibroblasts that were cultured at 32 °C. All cells were cultured on polystyrene‐treated plastic dishes (Corning) with EMEM media (ATCC) supplied with 15% FBS (Gibco), 100 units/mL penstrep.

### Firefly luciferase assay

One million fibroblasts were transfected with 5 μg of firefly luciferase plasmids and 0.1 μg Renilla luciferase plasmids. Cells were harvested 24 h after transfection and lysed in 200 μL passive lysis buffer (Promega). Firefly luciferase activity was read after 100 μL lysate was mixed with 100 μL LAR‐II substrate reagent, and then, 100 μL STOP&Glo was added to inactivate firefly luciferase and activate Renilla luciferase. The ratio of firefly to Renilla luciferase was taken to measure the frequency of mistranslation.

### Statistical analysis

Data from closely related taxa cannot be treated as statistically independent due to their common ancestry (Felsenstein, 1985). To correct for the influence of phylogenetic relatedness, an independent contrasts analysis was performed in Mesquite with the PDAP package (Maddison & Maddison, 2006). The time‐calibrated rodent phylogeny was kindly provided by Fabre *et al*. (2012). MLS contrasts were plotted against mistranslation contrasts, and the regression was analyzed by least squares.

## Funding

This work was supported by grants from US National Institutes of Health to VG, AS, ZZ, and VNG; Life Extension Foundation to VG and AS, Consejo Nacional de Investigaciones Científicas y Técnicas to FL, and NY Stem predoctoral fellowship to ZK.

## Conflict of interest

None declared.

## Supporting information


**Fig. S1** Translation fidelity does not correlate with species body mass.
