# Supplementary figures and images for: Translation fidelity coevolves with longevity
Source: Aging Cell. 2017 Jul 13;16(5):988–93. doi: 10.1111/acel.12628 (PMC5595694; doi:10.1111/acel.12628)

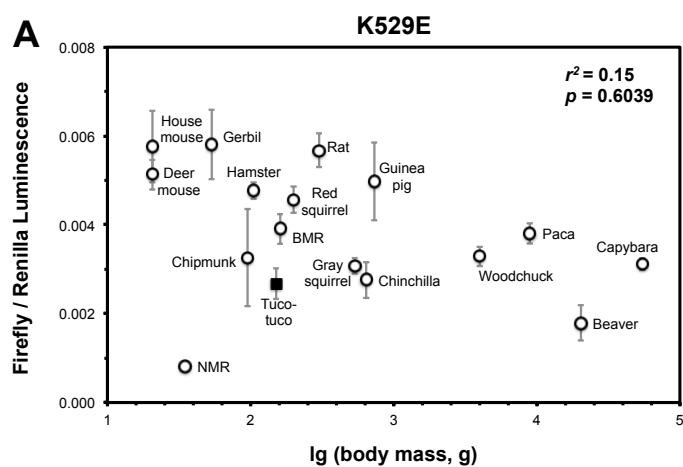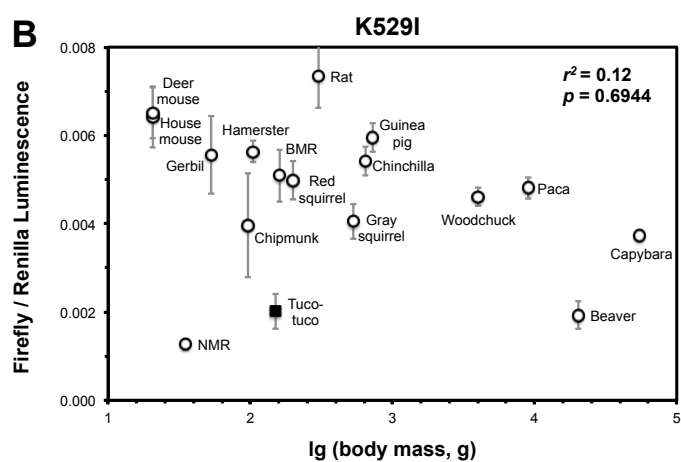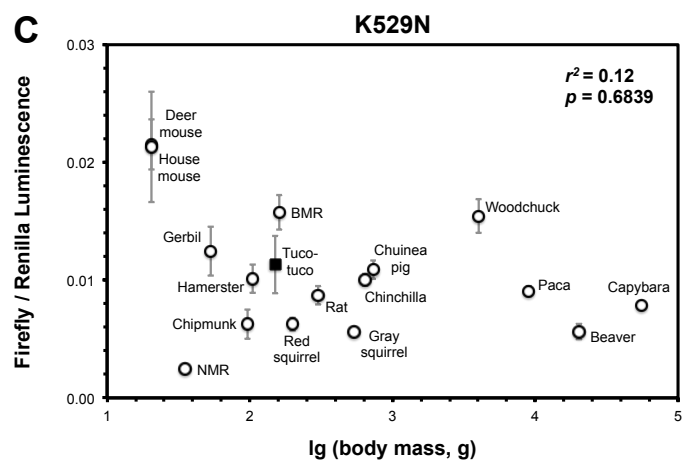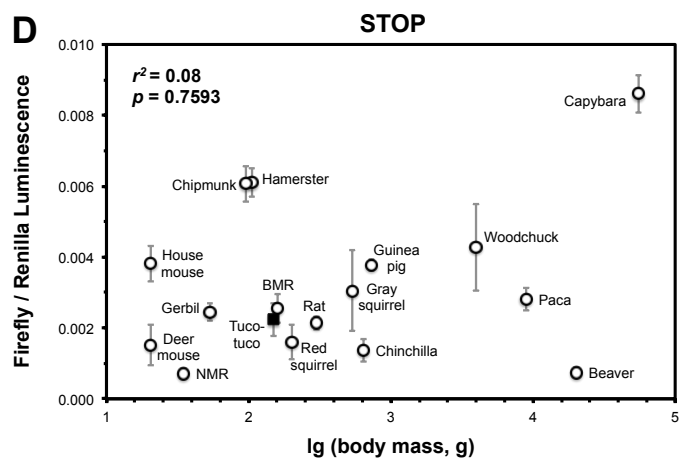

**Figure S1**

Supplement: Supplementary file 1 — Fig. S1 Translation fidelity does not correlate with species body mass. [file ACEL-16-988-s001.pdf]
