# Supplementary material for: Translation fidelity coevolves with longevity
Source: Aging Cell. 2017 Jul 13;16(5):988–93. doi: 10.1111/acel.12628 (PMC5595694; doi:10.1111/acel.12628)
Supplement: Supplementary file 2 [file ACEL-16-988-s002.doc]

**Figure S1. Translation fidelity does not correlate with species body mass.** Primary, low passage fibroblasts were cotransfected with the firefly reporter constructs described in Fig.  2 and Renilla luciferase. The error frequency was calculated as the ratio of firefly luciferase to the Renilla luciferase, transfection control, the lower the ratio, the higher the fidelity. Three independent cell lines were assayed each species, and each cell line was assayed in triplicate. Error bars show SEM. BMR, blind mole rat; NMR, naked mole rat. (B) Misincorporation at the first codon position (K529E) does not correlate with body mass. (C) Misincorporation at the second codon position (K529I) does not correlate with body mass. (D) Misincorporation at the third codon position (K529N) does not correlate with body mass. (E) Skipping of the STOP codon shows no significant correlation with body mass.
